# The helpful support of veno‐arterial extracorporeal membrane oxygenation to treat an exceptional systemic‐to‐pulmonary venous shunt: A case report

**DOI:** 10.14814/phy2.70847

**Published:** 2026-03-29

**Authors:** Ludovic van Delden, Matis Grundheber, Carole Looyens, Benjamin Assouline, Matthieu Papillard, Raphael Giraud, Karim Bendjelid

**Affiliations:** ^1^ Department of Anaesthesiology, Clinical Pharmacology, Intensive Care and Emergency Medicine Geneva University Hospitals Geneva Switzerland; ^2^ Department of Radiology and Medical Informatics Geneva University Hospitals Geneva Switzerland

**Keywords:** bronchogenic cyst, ECMO, endovascular therapy, mediastinal venous cavernoma, superior vena cava syndrome, systemic‐to‐pulmonary venous shunt

## Abstract

Systemic‐to‐pulmonary venous shunting is a rare cause of refractory hypoxemia. Its prompt recognition is crucial, as management requires not only etiological treatment but also the restoration of physiological venous return. We report the case of a 40‐year‐old man presenting with dyspnea, cervicothoracic edema, and hypoxemia. Imaging revealed a mediastinal mass compressing the superior vena cava (SVC), with associated subocclusive thrombosis in its lower segment and right pulmonary artery. Endobronchial ultrasound‐guided puncture and video‐assisted thoracoscopic marsupialization confirmed a benign bronchogenic cyst. Following anesthesia induction, the patient developed refractory hypoxemia requiring Veno‐Arterial Extracorporeal Membrane Oxygenation, chosen due to prohibitive obstruction of the SVC preventing veno‐venous access. Persistent hypoxemia prompted further investigations, revealing a right‐to‐left shunt through a mediastinal venous cavernoma connecting upper‐body venous return to the pulmonary veins. Mechanical thrombectomy of the SVC using the ClotTriever system restored venous patency, allowing ECMO weaning and full clinical recovery. This case highlights an exceptional systemic‐to‐pulmonary venous shunt. Successful management required a multimodal strategy integrating bronchoscopic, surgical, and endovascular interventions with ECMO support. Clinicians should consider extracardiac venous shunting as a rare cause of refractory hypoxemia and select the optimal ECMO configuration after careful evaluation of both hemodynamic status and vascular anatomy.

## 1 INTRODUCTION

Hypoxemia is a common clinical condition and includes a broad range of etiologies that may present significant diagnostic challenges. While some causes can be rapidly identified with basic investigations—allowing prompt treatment—refractory hypoxemia constitutes a life‐threatening emergency, requiring more advanced and invasive diagnostic and therapeutic strategies.

Right‐to‐left shunting, referring to the abnormal passage of systemic venous blood into the systemic arterial circulation, represents an uncommon cause of hypoxemia. From a pathophysiological point of view, these shunts are most frequently associated with congenital cardiac anomalies but may also be identified in otherwise healthy adults (Niggemann et al., 1987). In such cases, shunts most often reflect constitutional abnormalities—such as a patent foramen ovale or pulmonary arteriovenous malformations—and less commonly arise from functional acquired conditions.

Systemic‐to‐pulmonary venous shunting in the setting of superior vena cava (SVC) obstruction represents a rare and complex clinical entity. Most reported cases are secondary to malignancy or thrombosis due to medical devices (Rice et al., 2006), and result in the development of collateral venous channels between the systemic venous circulation and the pulmonary veins. Depending on the severity and anatomical extent of the obstruction, several distinct patterns of collateral venous drainage have been described (Kapur et al., 2010; Stanford et al., 1987), frequently involving the azygos system (Kapur et al., 2010), although cavernous transformation within the pulmonary circulation may occur (Kapur et al., 2010). Management focuses primarily on addressing the underlying cause and typically involves therapeutic anticoagulation and supportive care. In selected cases, direct endovascular intervention—either mechanical or chemical—or surgical approaches may be required.

The authors report a case of a benign bronchogenic cyst producing obstruction of the SVC with the development of a pericaval cavernous transformation and associated systemic‐to‐pulmonary venous shunting. The bronchogenic cyst was treated allowing restoration of the physiological systemic venous return via a multidisciplinary strategy combining bronchoscopic, surgical, and endovascular interventions, supported by Veno‐Arterial Extracorporeal Membrane Oxygenation (VA‐ECMO).

## 2 CASE REPORT

A 40‐year‐old man with no significant past medical history presented to the emergency department with a 4‐day history of mild dyspnea and headache, exacerbated in the supine position. He also reported pleuritic chest pain. On examination, he had sinus tachycardia (100–120 bpm), oxygen saturation of 86% on air, tachypnea (29/min), intercostal retractions, and a swollen, erythematous neck with a normal range of movement. Neurological examination was unremarkable.

Laboratory tests revealed mild systemic inflammation (CRP 40 mg/L; leukocytes 13,000/μL), hyponatremia (127 mmol/L), hyperkalemia (4.7 mmol/L), and markedly elevated D‐dimer (18,430 ng/mL). ECG and troponin levels were normal. Arterial blood gas showed respiratory alkalosis (pH 7.53; pO2 29.7 kPa; pCO2 3.67 kPa; lactate 1.3 mmol/L; HCO3 25.8 mmol/L).

**FIGURE 1:**
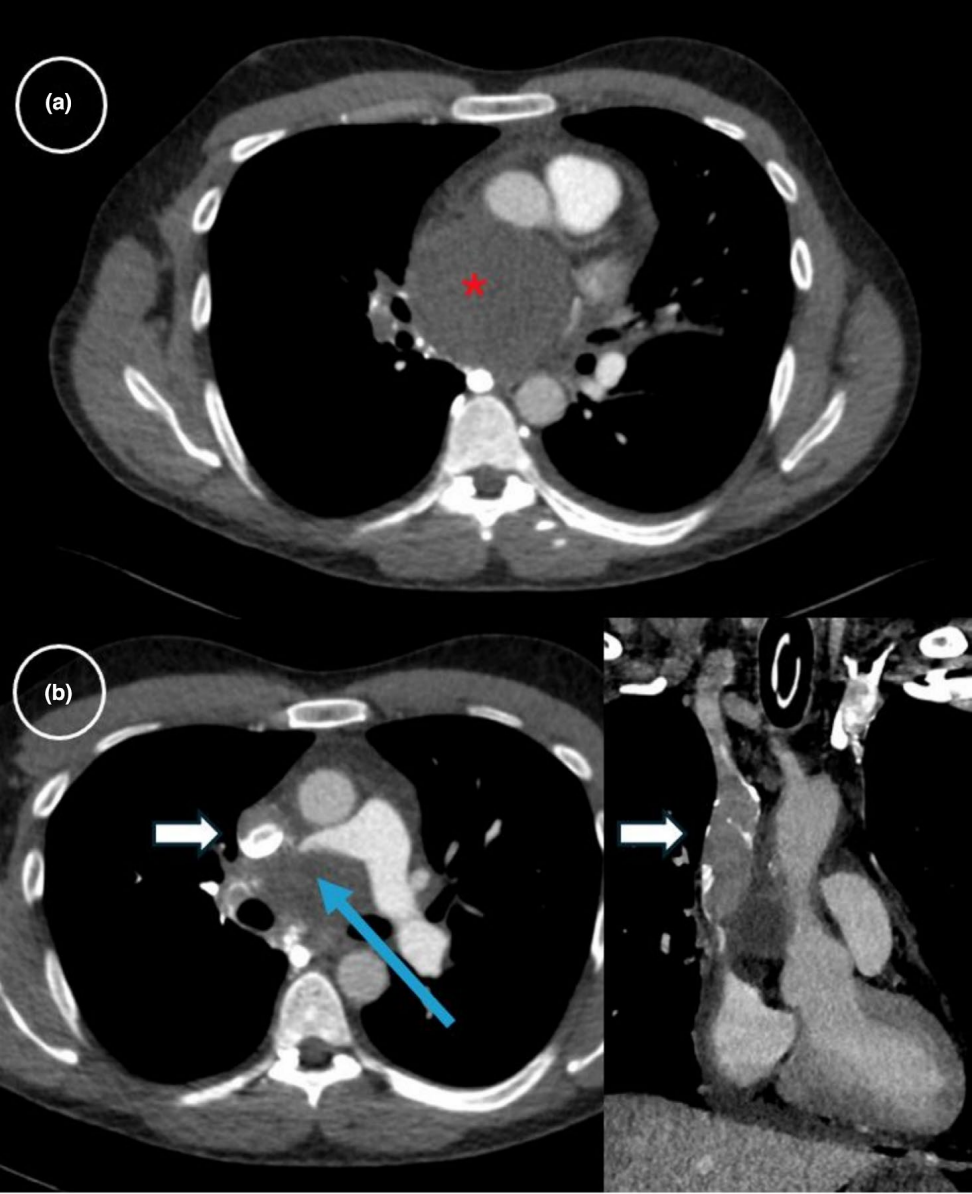
Contrast‐enhanced CT on admission showing (a) (*) a cystic mediastinal mass in the superior‐middle mediastinum, with (b) (white arrow) subocclusive thrombus in the superior vena cava and (blue arrow) near‐complete compression of the right pulmonary artery.

Contrast‐enhanced CT revealed a 7‐cm hypervascularized cystic mediastinal mass in the superior‐middle mediastinum, centered on the carina, causing mass effect on adjacent structures, including near‐complete compression of the right pulmonary artery, the esophagus, the upper left atrium, and SVC, which contained a subocclusive thrombus in its lower segment with recruitment of the azygos system (Figure 1). Imaging suggested a compressive bronchogenic cyst, although the radiological appearance wasn't entirely specific (hypervascularisation, heterogenous aspect). A cystic mediastinal lymphangioma or an esophageal duplication cyst were alternative diagnoses, considered less likely based on location and displacement of the esophagus. No mediastinal lymphadenopathy or pulmonary lesions were noted.

The patient was admitted to the intermediate care unit and started on therapeutic unfractionated heparin and supplemental oxygen (4–6 L/min). Empiric antibiotics (co‐amoxicillin) were given for suspected cyst infection; cultures remained sterile, and antibiotics were discontinued after 7 days.

Given the significant risk of right pulmonary artery ischemia and thrombus‐related complications (respiratory decompensation and/or major hemodynamic compromise), a diagnostic and therapeutic approach was considered via endobronchial ultrasound (EBUS) guided cyst puncture.

During the procedure, following anesthesia induction and instauration of positive pressure ventilation, the patient developed refractory hypoxemia (SpO_2_ 68%–74%) despite all reoxygenation maneuvers, without hemodynamic compromise, requiring initiation of femoro‐femoral veno‐arterial (VA) ECMO, as SVC obstruction precluded jugular veno‐venous (VV) approach. Partial cyst drainage was achieved, and the patient was admitted to the ICU.

Complete drainage was performed via video‐assisted thoracic surgery (VATS), with cyst marsupialization and drain placement; complete resection was not feasible due to extensive vascularization. Histology confirmed a bronchogenic cyst without malignant cells.

Despite pulmonary artery decompression, persistent severe hypoxemia led to ECMO weaning failure and prompted evaluation for an alternative etiology.

**FIGURE 2:**
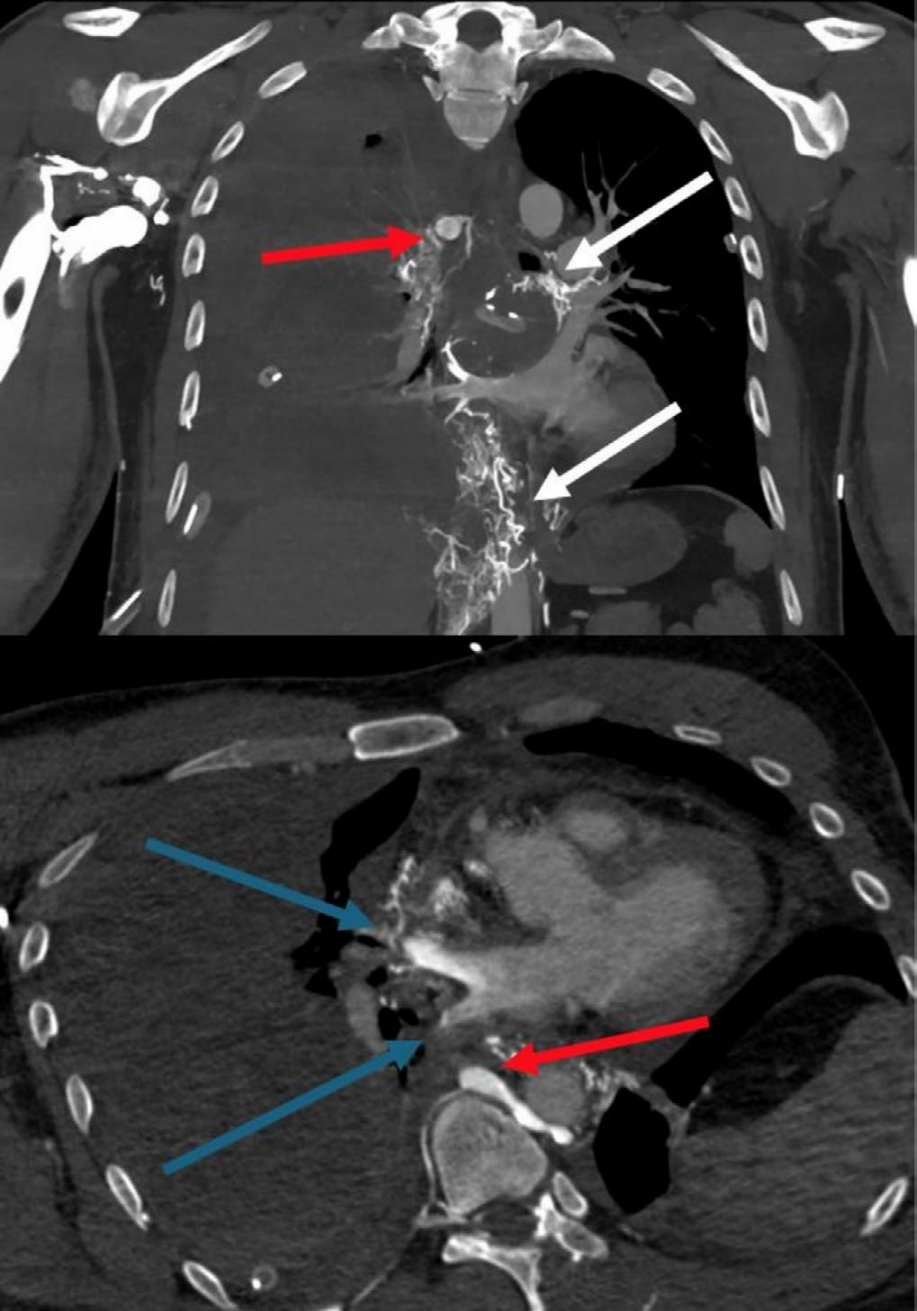
Contrast‐enhanced CT showing (red arrows) recruitment of the azygos system and (white arrows) a pericaval cavernous transformation connecting the upper limb venous system and the pulmonary veins, with (blue arrows) early venous opacification of the pulmonary vein through mediastinal anastomosis. Complete right lung atelectasis is secondary to hemothorax induced during the initial management strategy with therapeutic anticoagulation.

Contrast enhanced transesophageal echocardiography (bubble test) revealed a right‐to‐left shunt to the right superior pulmonary vein, which was visually quantified as a moderate to large shunt (between 20 and 50 microbubbles) (Lee & Oh, 2020). The anatomical origin could not be exactly delineated. Multidisciplinary CT review was therefore required and identified, besides the marsupialized cyst, a pericaval cavernous transformation connecting the upper limb venous system and the pulmonary veins, causing extra‐cardiac right‐to‐left shunting (Figure 2).

Conservative management with strict therapeutic anticoagulation was initiated given the high thrombotic burden and embolic risk. The course was complicated by an anticoagulation‐related hemothorax managed by thoracoscopy with clot evacuation, hemostasis, and chest drainage.

Subsequent anticoagulation appeared limited, yet surgery was excluded as a primary solution. The patient underwent mechanical thrombectomy using the ClotTriever system and balloon angioplasty of two stenotic segments located in the cavo‐atrial junction and the middle third of the subclavian vein. To minimize the theoretical risk of paradoxical embolism related to the right‐to‐left cavopulmonary venous shunt, a double Sentinel filter was placed via the right radial artery, protecting the brachiocephalic trunk and left carotid artery.

The clinical outcome was favorable, marked by restoring SVC patency, resolution of the right‐to‐left shunt, and successful VA‐ECMO weaning after 12 days of circulatory assistance. Follow‐up imaging subsequently showed near‐complete resolution of the SVC thrombosis. The patient was transferred from the ICU on day‐17 and discharged home 4 days later.

Follow‐up radiological assessment 2 months later demonstrated no cyst growth, resolution of the pericaval cavernous vascular abnormalities, and no evidence of impaired patency of the SVC system. Radiological monitoring was hereafter adjusted to a 6‐month interval.

## 3 DISCUSSION

Bronchogenic cysts are rare congenital malformations arising from abnormal budding or diverticulation of the primitive foregut during embryogenesis. These benign lesions, lined with respiratory epithelium, typically grow slowly due to mucus or fluid accumulation over time. Although frequently asymptomatic, they can cause complications such as infection, hemorrhage, airway compression, and more rarely, malignant transformation (Gross et al., 2023).

SVC syndrome is a well‐recognized yet potentially life‐threatening condition. The resulting increase in central venous pressure (CVP) in the upper body may lead to the recruitment of collateral channels that maintain systemic venous return to the right atrium, classically involving the azygos–hemiazygos system, intercostal, right thoracoepigastric, inferior epigastric, internal thoracic, and paravertebral veins (Kapur et al., 2010) ultimately draining into the inferior vena cava. In rare instances, however, marked elevation in CVP may induce aberrant functional venous channels between the systemic and pulmonary venous systems, creating an extracardiac right‐to‐left shunt and producing severe refractory hypoxemia.

In the present case, progressive compression of the right pulmonary artery and SVC by a benign bronchogenic cyst resulted in significant systemic venous congestion and hypertension, initiating a vicious circle of progressive thrombosis and rising systemic venous pressure, ultimately leading to the development of a pericaval cavernous transformation. This aberrant network of venous channels connecting the systemic venous circulation to the pulmonary veins, likely representing a congenital remnant reopened due to upper body venous pressure exceeding the downstream left atrial pressures, created an extracardiac right‐to‐left shunt, producing severe refractory hypoxemia and represents an exceptional manifestation of a bronchogenic cyst.

While surgical resection remains the gold standard for symptomatic or select asymptomatic bronchogenic cysts, EBUS fine‐needle aspiration—primarily a diagnostic technique in lung cancer staging—has gained traction as a temporizing intervention in patients who are not optimal surgical candidates (Anantham et al., 2011). For definitive management, minimally invasive approaches such as VATS have demonstrated excellent outcomes for cysts located within the mediastinum or lung parenchyma, offering reduced morbidity and recovery time compared with open thoracotomy (Gross et al., 2023).

Despite combined bronchoscopic and surgical decompression of the cyst—with restoration of right pulmonary artery flow—the right‐to‐left shunt as primary cause of hypoxemia persisted. Positive pressure ventilation increased intrathoracic and central venous pressures (Bendjelid, 2005) and, in the presence of persistent SVC obstruction due to organized thrombosis, sustained a pressure gradient favoring systemic‐to‐pulmonary venous shunting. Pulmonary venous pressure and flow are primarily determined by left atrial and left ventricular function, including ventricular relaxation. In the present case with a physiological left ventricular end‐diastolic pressure, systemic venous blood reaching the left atrium through the systemic‐to‐pulmonary shunt encountered a relative downstream pressure drop that facilitates forward flow.

By reducing venous return from both the superior and inferior vena cava, it further exacerbated SVC syndrome and increased shunt flow while simultaneously reducing inferior vena cava (IVC) return and overall transpulmonary blood flow. In addition, sedative agents used for general anesthesia may induce some degree of vasodilation and impair hypoxic pulmonary vasoconstriction, thereby worsening ventilation–perfusion mismatch and oxygenation. These combined effects increased the ratio of shunt flow to total transpulmonary flow, leading to worsening hypoxemia after anesthesia induction.

Relief of the RPA obstruction did not significantly increase transpulmonary flow because venous return to the right heart remained limited. In this context, transpulmonary flow depended primarily on IVC return, resulting in likely suboptimal right ventricular filling. The left pulmonary artery alone may have been sufficient to accommodate IVC flow during RPA obstruction, explaining the absence of major changes in ventilation–perfusion mismatch after RPA decompression. However, persistent SVC obstruction continued to limit additional venous return and maintained the functional shunt, allowing systemic venous blood from the upper body to bypass the pulmonary circulation and drain into the left heart, thereby sustaining hypoxemia despite redistribution of flow between the pulmonary arteries.

The resulting pathologic collateral circulation bypassing the obstruction of the SVC maintained sufficient blood flow to preserve left ventricular preload, albeit at the cost of severe hypoxemia necessitating temporary oxygenation support via extracorporeal membrane oxygenation. Although VV‐ECMO is the standard of care for refractory hypoxemia in critically ill patients (Tramm et al., 2013), it is exceptional for ECMO to serve as a bridge to the management of a systemic‐to‐pulmonary venous shunt associated with SVC obstruction. Notably, although VA‐ECMO is typically preferred in cases of refractory hypoxemia associated with right ventricular dysfunction and low transpulmonary flow (Grotberg et al., 2024), systemic arterial circulation of our patient was not compromised. Even though the right pulmonary artery was almost completely compressed, the flow through the left pulmonary artery maintained an adequate transpulmonary circulation to ensure left ventricular filling. VA‐ECMO was therefore chosen solely due to anatomical limitations that precluded jugular venous access. Femoro‐femoral VV‐ECMO was considered but carried an unacceptably high risk of recirculation and consequently reduced effective oxygenation (Pooth et al., 2025).

Definitive correction of the systemic‐to‐pulmonary venous shunt required recanalization of the SVC through either pharmacologic or mechanical thrombectomy, each carrying distinct risks. In our patient, the initial thrombolytic strategy resulted in hemorrhagic complications. As a safer alternative, the ClotTriever system—a mechanical thrombectomy device designed for “wall‐to‐wall” clot removal in vessels measuring 6–16 mm in diameter—was utilized.

Mechanical thrombectomy carries a potential risk of clot embolization; therefore, endovascular protection devices have been proposed to mitigate this complication, as in this case where a dual Sentinel filter was deployed due to the presence of the high flow right to left shunt (Haussig et al., 2020). Other aspiration thrombectomy devices are available, although comparative data remain limited, and device selection often depends on the interventionalist's experience and familiarity with specific systems. In this regard, mechanical thrombectomy restored both the patency of the SVC and the physiological transpulmonary flow. Consequently, the patient's systemic venous pressure and pressure gradient shunt decreased, leading to closure of collateral vessels and resolution of the right‐to‐left shunt allowing for ECMO weaning.

The present case underscores the crucial importance of a coordinated multidisciplinary strategy in managing complex pathophysiological conditions, requiring close collaboration among surgical and nonsurgical teams to mitigate risks and achieve optimal outcomes. The combined application of bronchoscopic, transthoracic, and endovascular interventions was essential in restoring normal anatomy and physiological hemodynamics. Moreover, clinicians should recognize ECMO as a viable supportive therapy in SVC syndrome complicated by severe refractory hypoxemia. Selecting the optimal ECMO configuration demands careful evaluation of both hemodynamic status and vascular anatomy, particularly if superior vena cava obstruction imposes a borderline configuration with the potential risk of recirculation.

## AUTHOR CONTRIBUTIONS

Conceptualization: LVD, MG, and KB. Data curation: LVD, MG, and MP. Formal analysis: LVD, MG, MP, and KB. Project administration: KB. Visualization: LVD, MG, and KB. Supervision: KB. Writing original draft: LVD and MG. Writing—review and editing: LVD, MG, CL, BA, MP, RG, and KB.

## FUNDING INFORMATION

This study was not supported by external funding.

## CONFLICT OF INTEREST STATEMENT

No potential conflict of interest relevant to this article.

## ETHICS STATEMENT

No institutional or ethics committee approval was required for this case report. Written informed consent was obtained from the patient for publication of this case report and any accompanying images.

## Supporting information


Appendix S1.

## Data Availability

Data sharing not applicable to this article as no datasets were generated or analyzed during the current study. The CARE checklist was submitted by the authors and is available as a Appendix S1.
