# Supplementary material for: The helpful support of veno‐arterial extracorporeal membrane oxygenation to treat an exceptional systemic‐to‐pulmonary venous shunt: A case report
Source: Physiol Rep. 2026 Mar 29;14(7):e70847. doi: 10.14814/phy2.70847 (PMC13140637; doi:10.14814/phy2.70847)
Supplement: Supplementary file 1 — Appendix S1. [file PHY2-14-e70847-s001.pdf]

CARE case report guidelines

CARE Checklist of information to include when writing a case report

(cc) BY-NC-ND

✓

Topic	Item	Checklist item description	Reported on Line
Title	1	The diagnosis or intervention of primary focus followed by the words "case report"	1 - 2
Key Words	2	2 to 5 key words that identify diagnoses or interventions in this case report, including "case report"	34 – 35
Abstract (no references)	3a	Introduction: What is unique about this case and what does it add to the scientific literature?	52 – 54 / 65
	3b	Main symptoms and/or important clinical findings	54 - 61
	3c	The main diagnoses, therapeutic interventions, and outcomes	57 – 58 / 61 - 65
	3d	Conclusion—What is the main "take-away" lesson(s) from this case?	65 - 69
Introduction	4	One or two paragraphs summarizing why this case is unique (**may include references**)	100 - 115
Patient Information	5a	De-identified patient specific information.	124
	5b	Primary concerns and symptoms of the patient.	125 - 126
	5c	Medical, family, and psycho-social history including relevant genetic information	124
	5d	Relevant past interventions with outcomes	124
Clinical Findings	6	Describe significant physical examination (PE) and important clinical findings.	126 - 129
Timeline	7	Historical and current information from this episode of care organized as a timeline	125 - 184
Diagnostic Assessment	8a	Diagnostic testing (such as PE, laboratory testing, imaging, surveys).	130 – 139 / 150 – 151 /157 – 159 / 161 – 164
	8b	Diagnostic challenges (such as access to testing, financial, or cultural)	148 – 156 / 158 – 159 / 160 – 161 / 167 - 172
	8c	Diagnosis (including other diagnoses considered)	139 – 143 / 159 / 165 - 166
	8d	Prognosis (such as staging in oncology) where applicable	159
Therapeutic Intervention	9a	Types of therapeutic intervention (such as pharmacologic, surgical, preventive, self-care)	144 – 146 / 150 – 151 / 154 – 158 / 167 – 170 / 172 - 176
	9b	Administration of therapeutic intervention (such as dosage, strength, duration)	147 / 176
	9c	Changes in therapeutic intervention (with rationale)	158 – 161 / 167 - 171
Follow-up and Outcomes	10a	Clinician and patient-assessed outcomes (if available)	177 - 180
	10b	Important follow-up diagnostic and other test results	181 - 184
	10c	Intervention adherence and tolerability (How was this assessed?)	–
	10d	Adverse and unanticipated events	152 – 156 / 160 – 161 / 168 - 170
Discussion	11a	A scientific discussion of the strengths AND limitations associated with this case report	203 - 209
	11b	Discussion of the relevant medical literature **with references**.	190 - 259
	11c	The scientific rationale for any conclusions (including assessment of possible causes)	190 - 250
	11d	The primary "take-away" lessons of this case report (without references) in a one paragraph conclusion	251 - 259
Patient Perspective	12	The patient should share their perspective in one to two paragraphs on the treatment(s) they received	–
Informed Consent	13	Did the patient give informed consent? Please provide if requested	Yes ☑ No ☐
